# Microscopic and Submicroscopic Asymptomatic Plasmodium falciparum Infections in Ghanaian Children and Protection against Febrile Malaria

**DOI:** 10.1128/IAI.00125-20

**Published:** 2020-09-18

**Authors:** Bright Adu, Quratul-ain Issahaque, Tracy Sarkodie-Addo, Selassie Kumordjie, Eric Kyei-Baafour, Caleb K. Sinclear, Sophia Eyia-Ampah, Eunice Owusu-Yeboa, Michael Theisen, Daniel Dodoo

**Affiliations:** a Department of Immunology, Noguchi Memorial Institute for Medical Research, University of Ghana, Legon, Ghana; b Department of Clinical Biochemistry and Immunology, Statens Serum Institut, Copenhagen, Denmark; c Centre for Medical Parasitology at Department of International Health, Immunology, and Microbiology and Department of Infectious Diseases, Rigshospitalet, University of Copenhagen, Copenhagen, Denmark; UC Davis School of Veterinary Medicine

**Keywords:** immunity, malaria, microscopic parasitemia, *Plasmodium falciparum*, submicroscopic parasitemia

## Abstract

Naturally acquired immunity to Plasmodium falciparum malaria is thought to be nonsterile and sustained by persistence of low-level parasitemia. This study assessed the association between baseline microscopic and submicroscopic asymptomatic P. falciparum infections and antimalarial antibody levels and whether these parasitemia modify protective associations between antibody levels and malaria in Ghanaian children. Healthy children (*N* = 973, aged 0.5 to 12 years) were recruited into a 50-week longitudinal malaria cohort study from January 2016 to January 2017. Baseline asymptomatic parasitemia were determined by microscopy (microscopic parasitemia) and PCR (submicroscopic parasitemia), and antibody levels against crude schizont antigens were measured by enzyme-limited immunosorbent assay (ELISA). Antibody levels, parasite diversity, and risk of malaria in the ensuing transmission season were compared among children who had baseline asymptomatic microscopic or submicroscopic or no P. falciparum infections. Of the 99 asymptomatic baseline infections, 46 (46.5%) were microscopic and 53 (53.5%), submicroscopic. Cox regression analysis adjusting for age group, sex and community found a strong association between both baseline microscopic (hazard ratio [HR] = 0.36, 95% confidence interval [95% CI] = 0.21 to 0.63; *P* < 0.001) and submicroscopic (HR = 0.22, 95% CI = 0.11 to 0.44; *P* < 0.001) asymptomatic parasitemia and a reduced risk of febrile malaria compared to those who were uninfected at baseline. Baseline asymptomatic submicroscopic parasitemia had a significant effect on associations between antischizont antibodies and protection against febrile malaria (*P* < 0.001; likelihood ratio test). The study found both baseline P. falciparum asymptomatic microscopic and more strongly submicroscopic infections to be associated with protection against febrile malaria in the ensuing transmission season. This could have important implications for malaria seroepidemiological studies and vaccine trials.

## Abstract (precis)

Naturally acquired immunity to Plasmodium falciparum malaria is thought to be nonsterile and sustained by persistence of low-level parasitemia. This study assessed the association between baseline microscopic and submicroscopic asymptomatic P. falciparum infections and antimalarial antibody levels and whether these parasitemia modify protective associations between antibody levels and malaria in Ghanaian children. Healthy children (*N* = 973, aged 0.

## INTRODUCTION

Plasmodium falciparum malaria remains a major cause of morbidity and mortality, particularly in pregnant women and children in sub-Saharan Africa, despite significant progress made by current control programs (1). In areas where malaria is endemic, the disease burden is reduced in adults due to an effective but nonsterile naturally acquired immunity (NAI) to the parasite through repeated exposure over time (2–5). The development of a robust NAI is thought to be slow and gradual, with initial acquisition of immunity against specific parasite strains until a broader repertoire of antibody and immune memory is attained to effectively control the plethora of different strains circulating in a typical area where malaria is endemic (6). Importantly, persistent exposure to the parasite seems necessary to maintaining NAI to malaria, which has been shown to wane in the absence of infective bites (7, 8).

P. falciparum infection may ultimately result in typical malaria symptoms, such as fever, chills, malaise, etc., which may develop into severe complications that are responsible for most fatalities associated with the disease. On the other hand, individuals may harbor P. falciparum parasites at different densities in their blood without the associated malaria symptoms (asymptomatic parasitemia), a phenomenon termed premunition, which is thought to help sustain NAI against malaria (4). In a recent systematic review, the association between asymptomatic P. falciparum infection and the risk of febrile malaria was reported to be different for different age groups and to be dependent on transmission intensity. In addition, multiclonal asymptomatic infections were associated with reduced risk of febrile malaria in older children in areas with high and moderate transmission (9), possibly due to the induction of a broader repertoire of antibodies. On the other hand, the contribution of submicroscopic parasitemia in perpetuating malaria transmission in regions where it is endemic has been thoroughly discussed (reviewed in reference 10). It is now well recognized that asymptomatic malaria infections could influence several important outcomes in both drug and immunological studies. For instance, in several drug and immunoepidemiological studies, baseline parasitemia is often considered a potential confounder of other outcome measures or protection against malaria and is typically controlled for in statistical models (11–16). Indeed, baseline parasitemia status is key among the identified variables of interest to take into account in the proposed guidelines for reporting Malaria Immuno-epidemiology Observational Studies (MIOS guidelines) (15). However, while the majority of studies have assessed baseline microscopic parasitemia, the potential effect of submicroscopic parasitemia on important outcomes such as antibody levels and protection against malaria in immunoepidemiological studies remain largely unknown. Here, we successfully assessed the association between microscopic and submicroscopic asymptomatic P. falciparum infections and antimalarial antibody levels and how parasitemia modifies antibody associations with the risk of febrile malaria in a longitudinal cohort study of Ghanaian children in a population with endemic malaria.

## RESULTS

### Participant characteristics and covariate association with baseline asymptomatic P. falciparum infection.

**FIG 1:**
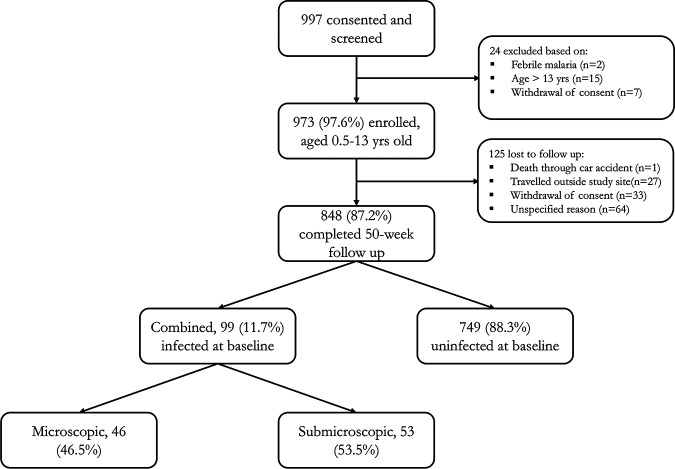
Subject enrollment and baseline infection characteristics. Children with baseline parasitemia that was high enough to be detected by conventional light microscopy were described as having microscopic infection. Children whose baseline parasitemia levels were so low that although microcopy detected no parasites, they were found by PCR to be infected were described as having submicroscopic parasitemia.

A total of 973 children (aged 0.5 to 13 years old) who met the inclusion criteria were enrolled out of the 997 screened. The excluded children were those who had febrile malaria (*n* = 2) or were older than 13 years (*n* = 15) or whose parents withdrew consent (*n* = 7) during screening. Of those enrolled, 848 (87.2%) completed the 50-week longitudinal follow-up. At baseline, a total of 99 (11.7%) children were infected with P. falciparum, of which 46 (46.5%) were detected by microscopy (microscopic) and the remaining 53 (53.5%) by PCR alone (submicroscopic) (Fig. 1). The remaining 749 (88.3%) carried no parasites at baseline either by microscopy or PCR determinations. All baseline infections were asymptomatic, with no fever or any other symptom of malaria. Asymptomatic baseline P. falciparum infections were more common in older children (>5 to 13 years old, microscopic = 8.3% and submicroscopic = 8.3%) compared to those who were younger (0 to 5 years old, microscopic = 1.8% and submicroscopic = 3.7%) (Table 1). Children with microscopic parasitemia had significantly (*P* = 0.0036) lower baseline hemoglobin (Hb) levels (mean [standard deviation], 10.5 [1.7] g/dl) than those with submicroscopic (11.3 [1.6] g/dl) or no P. falciparum infection (11.3 [1.5] g/dl) (Table 1). There were significant (*P* < 0.001) differences in the distributions of children who had either microscopic, submicroscopic, or no P. falciparum infection among the study communities but none for other covariates such as sex, sickle cell status, and bed net use (Table 1).

**TABLE 1:** Association between covariates and baseline asymptomatic P. falciparum infection status

Variable	Level	Uninfected (*n* = 749)	Submicroscopic^*a*^ (*n* = 53)	Microscopic^*a*^ (*n* = 46)	*P* value^*d*^
Age group (yrs) [no. (%)]	0–5	359 (94.5)	14 (3.7)	7 (1.8)	
	>5–13	390 (83.3)	39 (8.3)	39 (8.3)	<0.001
Hb mean (SD) (g/dl)^*b*^		11.3 (1.5)	11.3 (1.6)	10.5 (1.7)	0.0036
Sex [no. (%)]	Female	342 (87.9)	23 (5.9)	24 (6.2)	
	Male	407 (88.7)	30 (6.5)	22 (4.8)	0.64
Sickle cell status [no. (%)]^*c*^	Negative	592 (87.2)	45 (6.6)	42 (6.2)	
	Positive	25 (89.3)	2 (7.1)	1 (3.6)	0.85
Bed net use [no. (%)]	No	448 (87.3)	39 (7.6)	26 (5.1)	
	Yes	301 (89.9)	14 (4.2)	20 (6.0)	0.12
Community [no. (%)]	Adoteiman	274 (90.4)	15 (5.0)	14 (4.6)	
	Ayi-Mensah	136 (81.9)	15 (9.0)	15 (9.0)	
	Danfa	166 (92.7)	9 (5.0)	4 (2.2)	
	Ghana Flag	98 (97.0)	1 (1.0)	2 (2.0)	
	Kweiman	22 (91.7)	0 (0.0)	2 (8.3)	
	Otinibi	53 (70.7)	13 (17.3)	9 (12.0)	<0.001

a Submicroscopic refers only to children who were microscopy negative but were found to be infected by PCR analysis. Microscopic refers only to children who were found to be infected by microscopy.

b Missing data for 178 uninfected, 11 submicroscopic, and 7 microscopic.

c Missing data for 132 uninfected, 6 submicroscopic, and 3 microscopic.

d *P* values for hemoglobin (Hb) are for analysis of variance; for all other variables, chi-square *P* values are reported.

### Antimalarial drug intake prior to enrollment and submicroscopic parasitemia.

Antimalarial drug treatment history up to 30 days prior to testing has been associated with increased prevalence of submicroscopic parasitemia in previous studies (17); therefore, we assessed associations between self-reported antimalarial drug treatment and baseline infection status. Only orthodox antimalarial medication such as chloroquine and artemisinin-based drugs were included, while traditional herbal preparations were not considered. Overall, the distribution of individuals with a self-reported history of antimalarial drug intake within 2 weeks prior to enrollment was not significantly (χ^2^ = 1.42, *P* = 0.49) different among children with either microscopic, submicroscopic, or no infections at baseline. Nonetheless, the proportion of children who were reported to have taken antimalarial drugs 2 weeks prior to the baseline sampling was slightly higher in the group with submicroscopic infection (13.2%; 7/53) compared to those with microscopic (10.9%; 5/46) or no infection (8.7%; 65/749).

### Baseline infection status and antibodies against crude schizont antigens.

Isotype IgG and IgM antibody levels against P. falciparum crude schizont antigens were measured and compared among the different infection statuses at baseline in separate multiple linear regression analyses adjusting for age group, Hb, bed net use, sex, and community. There was no significant difference in baseline IgG levels between children who were uninfected and those with microscopic parasitemia (β = −0.04, 95% CI = −0.37 to 0.30; *P* = 0.83). However, children with submicroscopic parasitemia had significantly higher (β = 0.48, 95% CI = 0.13 to 0.82; *P* = 0.0065) IgG levels compared to those of the uninfected group (Table 2). There were no statistically significant differences in IgM levels between children with either microscopic or submicroscopic parasitemia and those in the uninfected group (Table 2). Since antibody data were log transformed, differences are on a natural log scale.

**TABLE 2:** Association between baseline parasitemia status and anti-schizont extract antibodies^*a*^

Antibody	Infection status	β	95% CI	*P* value	Adj. *R*^2^
IgG					
	Uninfected (*N* = 749)	1			0.15
	Microscopic (*N* = 46)	−0.04	−0.37, 0.30	0.83	
	Submicroscopic (*N* = 53)	0.48	0.13, 0.82	0.0065	
IgM					
	Uninfected (*N* = 750)	1			0.05
	Microscopic (*N* = 46)	−0.16	−0.57, 0.26	0.45	
	Submicroscopic (*N* = 53)	0.09	−0.33, 0.50	0.69	

a Multiple linear regression adjusting for age group, Hb, bed net use, sex, and community. Submicroscopic refers to children who were microscopy negative but were found by PCR analysis to be infected. Microscopic refers to children who were found by microscopy to be infected. Antibody data were log (base *e*) transformed to approximate normality.

### Febrile malaria episodes during the entire follow-up period.

Over the 50-week follow-up, 15.2% (129/848) of the study participants experienced at least one febrile malaria episode as defined by fever (measured axillary temperature of >37.5°C or fever reported) and any P. falciparum parasitemia determined by microscopy plus other clinical symptoms such as malaise, vomiting, joint pains, etc. These participants were classified as susceptible. Another 14.4% (122/848) were infected by P. falciparum at least once (microscopy and/or PCR detection of any parasitemia during monthly finger pricking) but did not develop fever or any malaria symptoms throughout the follow-up period. These participants were classified as protected. The final infection status of the remaining 70.4% (597/848) with respect to P. falciparum was classified as undefined since no parasite was detected in their blood by microscopy during either the monthly finger pricking or at any time point in the study period. This group was excluded from subsequent analysis in order to increase the power to clearly identify the effect of immunity in protection against febrile malaria as has been shown previously (18). P. falciparum detection by microscopy and not PCR was used in defining malaria cases in keeping to standard practice in health care facilities in Ghana.

### Association between baseline infection status and protection against malaria.

**FIG 2:**
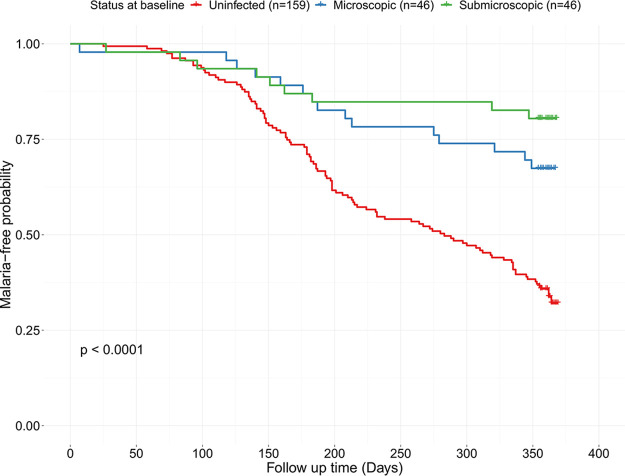
Kaplan-Meier estimates of malaria-free probability among children with different baseline infection statuses. There was a statistically significant difference in the risk of malaria (*P* < 0.0001; log rank test) among children in the different baseline infection groups (uninfected, *n* = 159; microscopic, *n* = 46; submicroscopic, *n* = 46). The Kaplan-Meier estimates of malaria-free probability showed that children with baseline microscopic or submicroscopic infections had a significantly reduced risk of malaria during the follow-up period compared to those with no baseline infection. Crosses denote censored observations.

Association between the three baseline P. falciparum infection status (i.e., uninfected, microscopic parasitemia, and submicroscopic parasitemia, respectively) and febrile malaria episodes in the ensuing transmission season was assessed using a Kaplan-Meier plot and log rank test (Fig. 2). There was a statistically significant (log rank *P* < 0.0001) difference in the probability of children in the different baseline infection status groups to remain free of malaria in the ensuing transmission season. Cox regression analysis adjusting for age group, sex, and community found a strong association between both baseline microscopic (hazard ratio [HR] = 0.36, 95% CI = 0.21 to 0.63; *P* < 0.001) and submicroscopic (HR = 0.22, 95% CI = 0.11 to 0.44; *P* < 0.001) asymptomatic parasitemia and a reduced risk of febrile malaria compared to that of those who were uninfected at baseline (Table 3).

**TABLE 3:** Association between baseline parasitemia status and risk of first febrile malaria episode during follow-up

Baseline infection status^*a*^	Statistic^*b*^		
	HR	95% CI	*P* value
Uninfected (*n* = 159)	1		
Microscopic (*n* = 46)	0.36	0.21, 0.63	<0.001
Submicroscopic (*n* = 46)	0.22	0.11, 0.44	<0.001

a Only study participants who had definitive exposure with parasitemia either at baseline or any time point during the follow-up were included in this analysis, hence the reduced numbers.

b Hazard ratios (HR) and 95% confidence intervals (95% CI) were calculated by Cox regression analysis adjusted for age group, sex, and community.

### Effect of baseline parasitemia on the association between antischizont antibodies and protection against febrile malaria.

**FIG 3:**
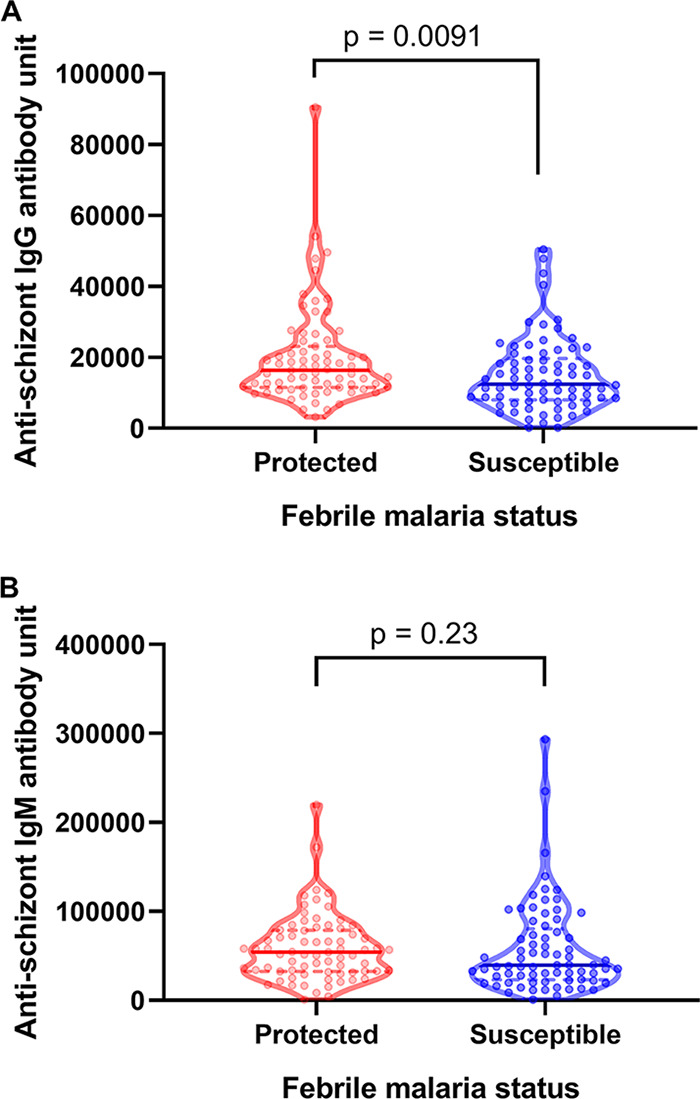
Comparison of antibody levels between protected and susceptible children. The violin plot shows raw IgG (A) and IgM (B) levels against schizont extract antigen for protected versus susceptible children. The upper and lower quartiles of each distribution are shown with broken lines above and below the median (solid line), respectively. Each point represents a child. Exact *P* values, which take into account ties among values, were calculated using the Mann-Whitney test (in GraphPad Prism 8.0.2) to compare the median antibody levels between protected and susceptible children.

Malarial antibodies have been shown to associate with protection against febrile malaria (16, 19). Overall, protected children in this study had significantly (*P* = 0.0091; Mann-Whitney test) higher median IgG levels compared to those of susceptible children; however, IgM levels were similar (Fig. 3). Having shown that baseline infection status influences the risk of febrile malaria in the ensuing transmission season, we next assessed the impact of microscopic and submicroscopic infections on the associations between antibodies and protection against malaria. In a logistic regression model adjusted for baseline submicroscopic and microscopic parasitemia, age groups, blood hemoglobin, bed net use, sex and community of residence, there was a significant association between baseline anti-schizont IgG (OR = 0.44, 95% CI = 0.20 to 0.99; *P* = 0.046) and protection against febrile malaria but not for IgM (*P* = 0.36) (Table 4). The likelihood ratio test showed a significant effect of baseline submicroscopic parasitemia on the associations between febrile malaria for both IgG (*P* = 0.00064) and IgM (*P* < 0.0001) (Table 4).

**TABLE 4:** Association between anti-schizont extract antibodies and protection against malaria

Antibody	Statistic^*a*^			
	OR	95% CI	Adj. *P* value	LR test *P* value
IgG	0.44	0.20, 0.99	0.046	0.00064
IgM	0.77	0.44, 1.34	0.36	<0.0001

a The odds ratio (OR) and 95% confidence intervals (95% CI) with adjusted (adj.) *P* values were calculated by logistic regression analysis adjusted for baseline submicroscopic and microscopic parasitemia, age groups, blood hemoglobin, bed net use, sex, and community of residence. The binary outcome variable used in the logistic regression analysis was febrile malaria status categorized at two levels (protected children [*n* = 122] and susceptible children [*n* = 129]). The likelihood ratio (LR) test result compares the adjusted model to a model which excludes baseline submicroscopic parasitemia and thereby tests its effect on the adjusted model.

## DISCUSSION

We assessed the risk of febrile malaria in a longitudinal cohort study involving Ghanaian children who had either microscopic or submicroscopic asymptomatic P. falciparum infections prior to the transmission season (i.e., baseline) in an area where malaria is endemic in southern Ghana. The overall low prevalence of asymptomatic parasitemia at baseline was expected, since in Ghana, malaria is currently more prevalent in the middle and northern sectors than in the south, where only pockets of areas with high transmission are seen (20). A greater proportion of older children harbored asymptomatic parasites (either microscopic or submicroscopic) compared to those who were younger, which may be indicative of naturally acquired immunity. This is consistent with reports showing increased asymptomatic parasitemia prevalence with age and a later decline in adulthood (17, 21–24). Several factors may account for this observation, including increased use of long-lasting insecticide-treated nets in the younger age group children (25) and the higher likelihood of them getting earlier treatment once infected compared to that of older children. Conversely, a comparatively stronger NAI in older children may explain their ability to tolerate parasitemia in the absence of febrile malaria symptoms. None of the children enrolled were anemic; however, children with microscopic asymptomatic infections had a lower Hb level compared to that of those with submicroscopic parasites or no infection, consistent with previous studies (17, 26, 27). The rupture of matured schizont stage parasites or their premature phagocytosis and/or destruction by the reticuloendothelial system may contribute to a decreased blood Hb level (28, 29).

Fever in children aged 2 to 10 years but not in those <2 years old or adults was attributed to both microscopic and submicroscopic parasitemia in Uganda (30). Here, none of the infected children at baseline were febrile at the time of sampling, and they did not show any clinical symptoms associated with malaria, suggesting that these asymptomatic parasites may be contributing to nonsterile immunity (premunition) observed in malaria (4) at least in some of the children. The impact of asymptomatic P. falciparum infection on the risk of malaria is not well understood and may vary across different transmission settings and be dependent on different parasite and host factors (31–33). Indeed, while some have reported increased risk of symptomatic malaria with asymptomatic parasitemia carriage (30, 34, 35), others have found asymptomatic infections to be rather protective (36–39), consistent with our data. It has also been reported that asymptomatic parasitemia treatment did not increase the risk of clinical malaria upon reinfection (36). In general, most semi-immune individuals in areas where malaria is endemic carry asymptomatic parasitemia, and this is thought to be involved in the development of naturally acquired immunity against malaria (4, 5, 32, 40). It is likely that, in areas where asymptomatic parasitemia has been associated with increased risk of malaria, the former may represent early replicating parasites that later resulted in febrile malaria (30). On the other hand, immunity to malaria is strain specific (3, 41), and infections with new parasite strains different from those causing chronic asymptomatic parasitemia may result in symptomatic malaria if prevailing antibodies lack cross-reactivity against the new strains (3, 40). Our ongoing studies involve using next-generation sequencing to assess whether asymptomatic strains were different from symptomatic infections in the same cohort.

Increased prevalence of submicroscopic parasitemia 30 days post antimalarial drug treatment in previously parasitemic individuals have also been reported (17). Here, we found no statistically significant association between submicroscopic parasitemia prevalence and self-reported antimalarial drug treatment 2 weeks prior to the baseline sampling; however, this should be interpreted with caution due to potential subjectivity of self-reported data. Nonetheless, there was a trend showing a slightly higher proportion of individuals with self-reported history of antimalarial drug treatment prior to baseline sampling in the submicroscopic infection group compared to the others, in agreement with previous findings (17).

Traditionally, IgM is thought to be an early response antibody that is subsequently replaced by IgG during infections (42, 43). In contrast, recent studies have shown that in malaria, IgM responses are long-lived and may persist even in the absence of reinfections and that IgM shows similar decay kinetics to those of IgG (44, 45). This may explain why we observed no significant difference in IgM levels among the different groups of children studied.

Both children with baseline asymptomatic submicroscopic and microscopic P. falciparum infections were protected against febrile malaria in the ensuing transmission season compared to those who had no baseline parasitemia in the current study. However, the magnitude of protection observed was higher in children with baseline submicroscopic (78%) than in those with microscopic (64%) parasitemia. This may be due to perhaps comparatively more of the baseline asymptomatic microscopic parasitemia later developing into febrile episodes. However, it is not known whether there exists a minimum threshold of parasitemia that is required to sustain functional protective nonsterile immunity against malaria. Naturally acquired protective immunity against malaria is thought to be sustained through persistent exposure to different parasite strains in a population (3, 4, 6) in order to build a broader repertoire of antibodies. Furthermore, the higher levels of baseline antimalarial IgG in the submicroscopic infection group may have contributed to their greater protection. It was evident that both baseline microscopic and submicroscopic parasitemia significantly modify the protective effects of malaria-specific antibodies. Taken together, findings from this study supports the premunition hypothesis and further underscore the importance of submicroscopic parasitemia naturally acquired protective immunity against febrile malaria. It further indicates that, in studies where baseline parasitemia is of interest as a potential confounder to other measures of protection, both microscopic and submicroscopic parasites must be accounted for in such analyses.

In conclusion, the study found baseline P. falciparum asymptomatic microscopic and, more strongly, submicroscopic infections to be associated with protection against febrile malaria. These findings have important implications for studies where the confounding effect of baseline asymptomatic malaria infections need to be considered such as in vaccine trials and seroepidemiological studies.

## MATERIALS AND METHODS

### Ethics statement.

The study was reviewed and approved by the Institutional Review Board of the Noguchi Memorial Institute for Medical Research (NMIMR) of the University of Ghana, Accra, Ghana, and by the Ghana Health Service Ethics Committee of the Ministry of Health, Ghana. Written informed consent was given by the parents and guardians of all children before they were enrolled into the study.

### Study area and population.

The study was a conducted in six periurban communities, of which four, Danfa, Kweiman, Adoteiman and Otinibi, were in the La-Nkwantanang-Madina Municipal assembly (latitude, 5.677778; longitude, −0.16694) and two, Ayi-Mensah and Ghana Flag were in the Ga-East Municipal Assembly (latitude, 5.731790; longitude −0.204310), all in the Greater Accra Region of Ghana. These communities were selected due to proximity to each other and to a common health facility for easy tracking of malaria cases, as well as proximity to the NMIMR to ensure that samples were processed in time. The study area has different ethnic groups of Ghana—mainly Ga, Akan, Ewes, and Hausa—living together in within the same communities. The area is within the coastal savannah zone of Ghana, and rainfall pattern is bimodal with a major peak around May to July and a minor peak from September to November. Malaria transmission mirrors the rainfall pattern and peaks during or immediately after the rainy seasons, with P. falciparum being the main malaria parasite causing majority of infections. The main health facility serving the study communities is the Danfa Health Centre (DHC), which was also the designated health facility for the study where all study participants were encouraged to attend as their first point of care when ill. Medical cases beyond the capacity of the DHC are referred to bigger hospitals such as the Dodowa, Pentecost, 37 Military, and Ridge hospitals, all within the Greater Accra Region of Ghana.

### Study design and sampling.

A total of 997 children whose parents or guardians consented for their inclusion in the study were screened, and 973 between the ages of 0.5 to 12 years were enrolled into a 50-week longitudinal cohort study prior to the malaria season in January and February 2016. Children aged <0.5 or >13 years or with a positive malaria rapid diagnostic test (RDT) [First Response Malaria Ag(pLDH/HRP2) Combo RDT, catalog no. I16FRC30] and/or P. falciparum parasitemia in addition to fever (axillary temperature, >37.5°C) or whose parent/guardian withdrew consent during the screening process were excluded from enrollment. Those without fever at baseline were not tested with malaria RDT. Children presenting with any chronic medical condition were referred to the DHC for treatment and were also excluded. At enrollment (baseline), each study participant was assigned a unique study identifier and a questionnaire was completed to capture the clinical/medical history and demographic data of participants. Axillary body temperature was measured, and about 3 ml of venous blood (children ≥ 2 years) or 0.5 to 1 ml of finger-prick blood (children < 2 years) samples were collected into EDTA anticoagulant tubes for immunological assays. Blood hemoglobin level measurement by Hemocue-Hb 201 (Angelhom, Sweden) and sickle cell status (sodium metabisulphite test) were determined. In addition, blood slides for malaria parasitemia estimation by light microscopy and filter paper (3MM; Whatman Int. Ltd., England) dried blood spots (DBS) for parasite genetic diversity studies were obtained. There was weekly active follow-up in which trained field assistants visited the home of each child to measure axillary body temperature and completed a morbidity questionnaire with the help of the parent/guardian on the child’s wellbeing. Children found to be unwell or with measured or reported fever were referred to the DHC, where DBS and blood smears for malaria parasite studies were collected and treatment administered by the medical staff in the facility. Passive surveillance was throughout the study period when the parent/guardian visited the DHC outside the scheduled field assistant’s weekly visits when the child was unwell, which they were encouraged to do. DBS and blood film samples were also obtained during such visits. All malaria cases were treated with artemisinin-based combination therapy, which was the standard treatment according to the Ghana Health Service guidelines at the time of the study. Once every month, DBS and blood smears for parasite detection and genetic diversity studies were obtained from each study participant by finger pricking. Participants who missed 3 consecutive monthly finger pricks were excluded from the final analysis.

### Sample processing and storage.

All samples were processed at the Immunology Department of the NMIMR, which is only about a 40-min drive from the study site. Blood samples were centrifuged using a refrigerated centrifuge (Sakuma Mfg. Co. Ltd., Tokyo, Japan) at 2,000 rpm for 10 min, and the plasma and buffy coat were separated and stored at −40°C until use. The DBS, packed individually in resealable zipper lock bags, were kept at −20°C until use. Blood smears were stained with 10% Giemsa for Plasmodium parasite examination by light microscopy as described previously (46).

### Schizont extract enzyme-linked immunosorbent assay.

P. falciparum NF54 schizont extract was obtained as previously described (47), and the enzyme-limited immunosorbent assay (ELISA) protocol was as described elsewhere (16) with slight modifications. Briefly, flat-bottomed 96-well microtiter plates (Nunc MaxiSorp, Denmark) were coated with 5 μg/ml crude schizont antigen in 1× phosphate-buffered saline (PBS; pH 7.4) at 100 μl/well and incubated overnight at 4°C. An ELISA was performed with plasma samples diluted at 1:500 in sample dilution buffer (PBS with 1% milk powder, 0.1% Tween 20, and 0.02% Na-azide) and added at 100 μl/well in duplicates. Isotype IgG and IgM were detected in respective assays by adding 100 μl/well of either goat anti-human IgG (H10307) (1:3,000) or IgM (31415) (1:5,000) conjugated to horseradish peroxidase (Invitrogen Corporation, Camarillo, CA). A standard reference curve was obtained by a 3-fold serial dilution of pooled hyperimmune plasma included on each plate. Optical density values for the test samples were transformed to antibody units based on the standard reference curves using a Microsoft Excel-based 4-parameter logistic curve-fitting application (ADAMSEL b040; Ed Remarque, 2009).

### DNA extraction.

DNA from stored DBS was extracted by the Chelex method (48) with slight modifications. Briefly, a sterile punch was used to cut out about a 3-mm square piece of a blood blot on filter paper into labeled 1.5-ml microcentrifuge tubes. To prevent cross-contamination between samples, the punch was washed in 5% NaOH, 10% bleach, and distilled water and wiped thoroughly after each cut. A volume of 1 ml of sterile 1× PBS and 50 μl of 10% saponin solution added to the tubes and gently vortexed and incubated at 4°C overnight. The tubes were centrifuged at 14,000 rpm for 30 s and the supernatant discarded after which 1 ml of 1× PBS without saponin was added and inverted several times and incubated at 4°C for 30 min. Tubes were centrifuged as above, and the supernatant was discarded, leaving the filter paper. Sterile distilled water (100 μl) was added and the contents centrifuged as above, and the supernatants were discarded by pipetting. A volume of 70 μl sterile distilled water was added followed by 30 μl of 20% wt/vol Chelex-100 resin suspension in deionized water. The tubes were then vortexed and incubated at 95°C using a Techne heating block (Bibby Scientific Ltd., UK) for 10 min with vortexing at 2-min intervals. After incubation, the tubes were centrifuged at 14,000 rpm for 6 min and then the supernatant (containing DNA) was transferred into prelabeled 96-well plates for use as the template in PCRs. The concentration of the genomic DNA was determined using a NanoDrop 2000C spectrophotometer (Labtech International, UK) and stored at −20°C until use.

### PCR detection of P. falciparum parasites.

All of the samples from baseline already screened by microscopy were also assessed for parasitemia using PCR. This allowed for identification of individuals with submicroscopic parasitemia alone who were defined as being microscopy negative but PCR positive for P. falciparum infection. P. falciparum detection by PCR was carried out with primers targeting a 276-bp fragment of the 18S rRNA gene as previously described (49) with modifications to the reaction and cycling conditions. The reactions were performed in a 20-μl final volume containing 20 to 40 ng template DNA, 4 μl 5× PCR buffer (100 mM Tris-HCl [pH 8.9] at 25°C, 110 mM KCl, and 9 mM MgCl_2_), 0.25 mM mixture of each deoxynucleoside triphosphate (dNTP), 0.25 mM each primer (Inqaba Biotec Co. Ltd., Ghana), and 0.0375 U of One*Taq* Hot Start DNA polymerase (New England Biolabs, Ipswich, MA). All reactions had a genomic DNA from a culture of P. falciparum laboratory strain 3D7 as a positive control. The PCR cycling (Eppendorf, Hamburg, Germany) conditions were an initial activation step of 94°C for 15 min, followed by 35 cycles of denaturation for 30 s at 94°C, annealing for 45 s at 54°C, and extension for 45 s at 68°C. The final extension was carried out for 5 min, also at 68°C. The PCR products were visualized (Bio-Print gel documentation system; Vilber Lourmat, France) by electrophoresis on 2% agarose with ethidium bromide staining.

### Statistical analysis.

Study participants were categorized into three groups based on baseline infection status as either uninfected or having either submicroscopic or microscopic infections. Submicroscopic infections were defined as infections that could not be detected by microscopy and were only detected by PCR targeting the P. falciparum 18S rRNA gene. Proportions of study participants distributed among age groups, sex, sickle cell status, bed net use, and communities were compared for each variable between the three categories by the chi-square test. Hb was normally distributed and was compared among the groups by analysis of variance (ANOVA). The Mann-Whitney test, which considers ties among values, was used in calculating exact *P* values to compare the median antibody levels between protected and susceptible children. Antibody data were log (base *e*) transformed to approximate normality and differences compared among the groups with the uninfected group as the reference in a multiple linear regression adjusting for age group, Hb, bed net use, sex, and community. The association between antischizont antibody levels and protection against malaria was assessed by logistic regression analysis, adjusting for covariates. A likelihood ratio test was used to estimate the impact of baseline parasitemia status on associations between antibody levels and protection against malaria. The associations between baseline infection status and time to first malaria episode in the 50-week follow-up was assessed by Kaplan-Meier plots, and log rank statistics and hazard ratios were calculated by Cox regression analysis. All data analyses were performed in R v3.6.0 (50) and GraphPad Prism 8.0.2. *P* values of <0.05 were considered statistically significant.
